# Supplementary material for: A Cooperative Model for Symmetric Ligand Binding to Protein Fibrils
Source: bioRxiv. 2025 Jan 30:2025.01.29.635590. Preprint. [Version 1] doi: 10.1101/2025.01.29.635590 (PMC11838254; doi:10.1101/2025.01.29.635590)
Supplement: 1 [file NIHPP2025.01.29.635590v1-supplement-1.pdf]

bioRxiv preprint doi: https://doi.org/10.1101/2025.01.29.635590; this version posted January 30, 2025. The copyright holder for this preprint (which was not certified by peer review) is the author/funder, who has granted bioRxiv a license to display the preprint in perpetuity. It is made available under aCC-BY 4.0 International license.

Supplemental Information for

A Cooperative Model for Symmetric Ligand Binding to Protein Fibrils

Matthew S. Smith[1,2]*, William F. DeGrado[1,3], Michael Grabe[1,3], Brian K. Shoichet[1]

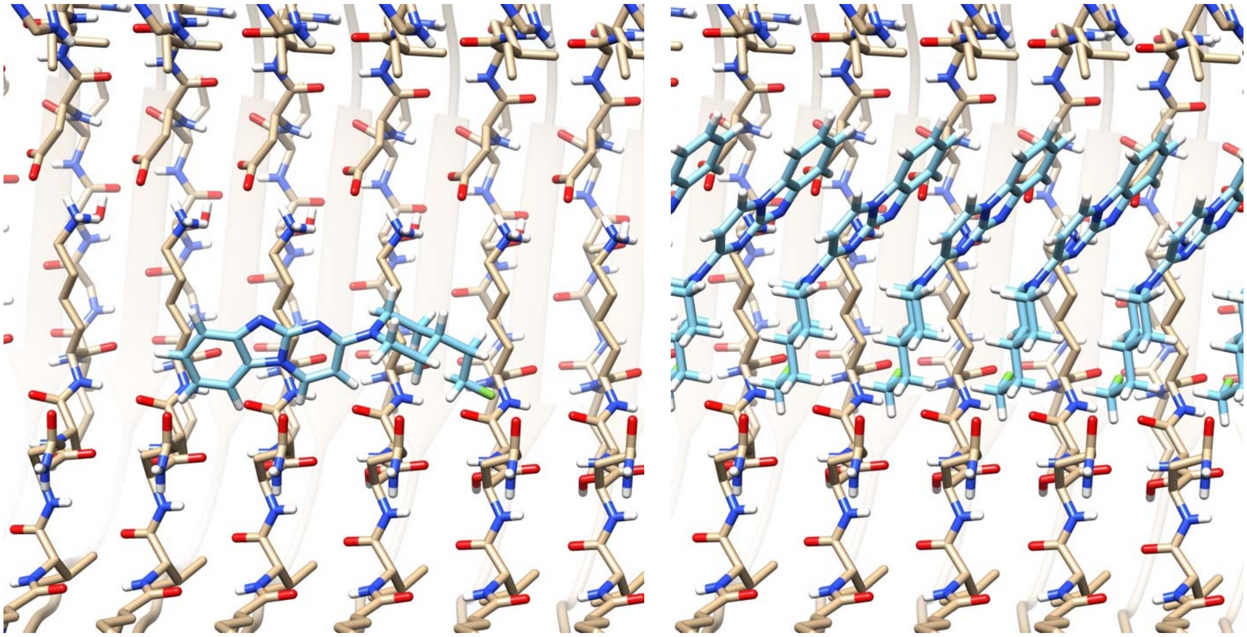

Figure S1. Docking the molecule GTP-1 with (left) and without (right) a stacking symmetry requirement shows how the molecule's interactions with the AD PHF tau fibril change.

bioRxiv preprint doi: https://doi.org/10.1101/2025.01.29.635590; this version posted January 30, 2025. The copyright holder for this preprint (which was not certified by peer review) is the author/funder, who has granted bioRxiv a license to display the preprint in perpetuity. It is made available under aCC-BY 4.0 International license.

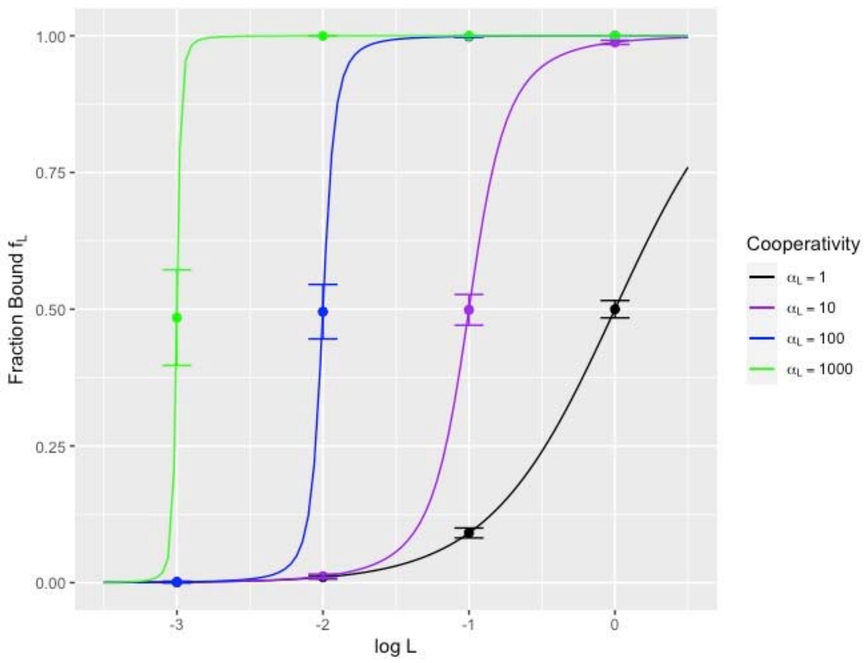

Figure S2. Theoretical binding curves from a saturation experiment for binding to an infinite symmetric fibril compared to MCMC simulations run for a fibril of length $N = 1000$ at different (L, α_L) values. The MCMC simulations comprised modeling the fibril of a 1000-bit binary string, where each proposed step was flipping a random bit with acceptance probability given by the Boltzmann factors. Each (L, α_L) pair had 10 independent MCMC simulations run at different seeds for the initial configuration and pseudo-random number generator. We ran 1 million steps of equilibration after initializing each simulation without collecting data to equilibrate. After equilibration, we collected data (the binary string) for 10 billion simulation steps. Points on the graph denote averages and error bars denote standard deviations of the aggregate counts across all 10 simulations seeds. Using $L = \frac{[L]}{K_D}$.

bioRxiv preprint doi: https://doi.org/10.1101/2025.01.29.635590; this version posted January 30, 2025. The copyright holder for this preprint (which was not certified by peer review) is the author/funder, who has granted bioRxiv a license to display the preprint in perpetuity. It is made available under aCC-BY 4.0 International license.

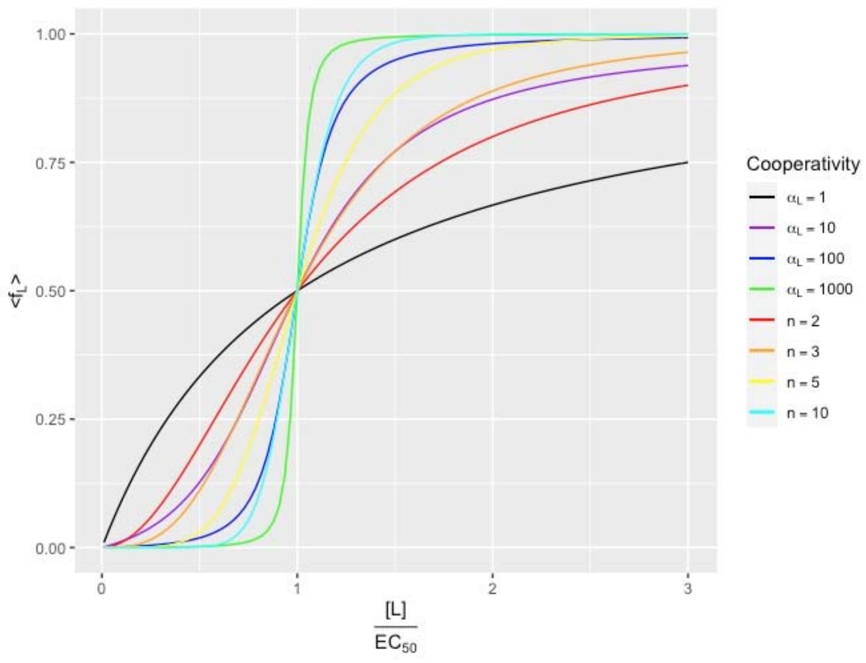

Figure S3. Comparing the cooperativity between the infinite symmetric fibril model in a saturation experiment (for different cooperativity factors α_L) to the empirical Hill Equation

$$f = \frac{[L]^n}{K_D + [L]^n} \#(S1)$$

(for different Hill Coefficients n). For the infinite symmetric fibril, $EC_{50} = \frac{K_D}{\alpha_L}$, by EQ 5 in the main text, and K_D has units of $\mathrm{mol} / \mathrm{L}$. The Hill Equation gives the fraction bound with no ligand depletion for the reaction $R + nL \rightleftharpoons RL_n$ (for receptor R and ligand L), so K_D has units of $(\mathrm{mol} / \mathrm{L})^n$, and $EC_{50} = \sqrt[n]{K_D}$.

bioRxiv preprint doi: https://doi.org/10.1101/2025.01.29.635590; this version posted January 30, 2025. The copyright holder for this preprint (which was not certified by peer review) is the author/funder, who has granted bioRxiv a license to display the preprint in perpetuity. It is made available under aCC-BY 4.0 International license.

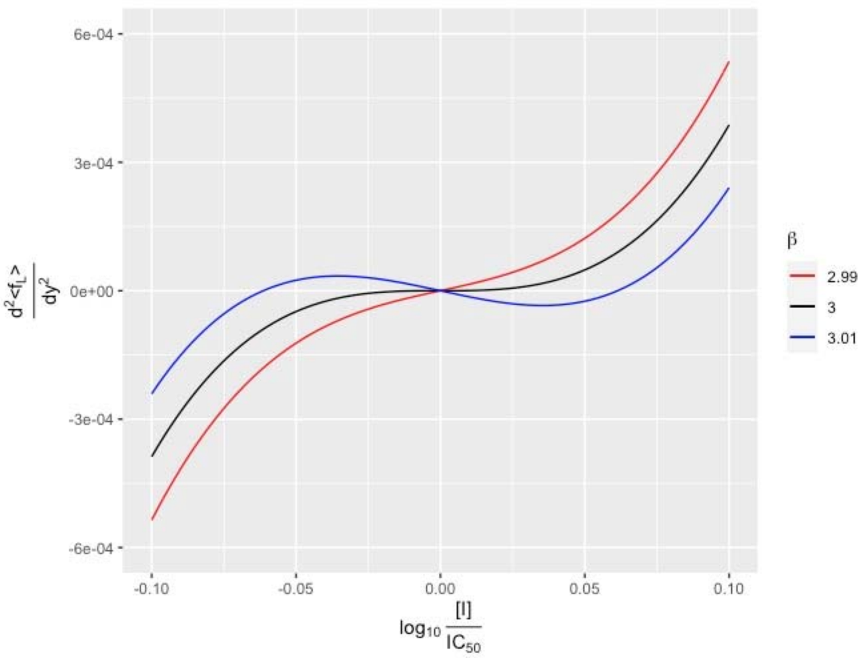

Figure S4. Showing the transition from monosphasic to biphasic competition binding curves at $\beta = 3$ by plotting EQ S36.

bioRxiv preprint doi: https://doi.org/10.1101/2025.01.29.635590; this version posted January 30, 2025. The copyright holder for this preprint (which was not certified by peer review) is the author/funder, who has granted bioRxiv a license to display the preprint in perpetuity. It is made available under aCC-BY 4.0 International license.

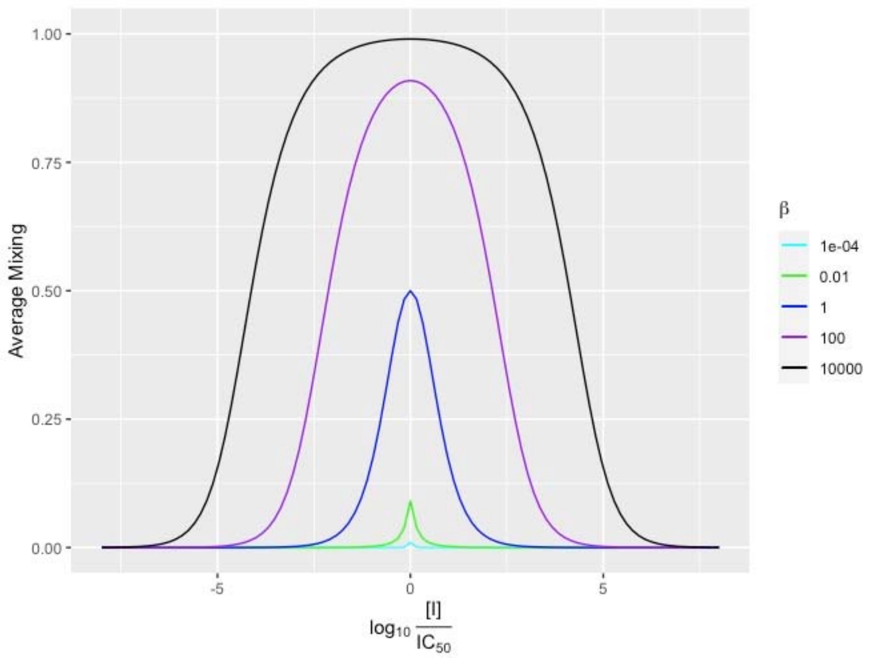

Figure S5. Average mixing between sites bound with radioligand and sites bound with inhibitor in the competition assay when all sites are bound with something as given by EQ S43.

bioRxiv preprint doi: https://doi.org/10.1101/2025.01.29.635590; this version posted January 30, 2025. The copyright holder for this preprint (which was not certified by peer review) is the author/funder, who has granted bioRxiv a license to display the preprint in perpetuity. It is made available under aCC-BY 4.0 International license.

Supplemental Methods: Transfer Matrix for Equilibrium Isotherm

We will begin with deriving the equilibrium isotherm for the radioligand saturation experiment. A fibril with N sites, each of which can be filled with a radioligand or empty, has 2^N possible configurations. Each configuration has an equilibrium probability proportional to its Boltzmann weight:

$$\mathbb{P}(config) \propto \exp\frac{-\mathcal{H}}{k_B T}, \#(S2)$$

where k_B is Boltzmann's constant, T is the absolute temperature, and $\mathcal{H}$ is the energy (Hamiltonian) of the configuration. The system gets an added energy $\Delta G_L = -k_B T \ln\frac{[L]}{K_D}$ for each site occupied with radioligand, for free ligand concentration $[L]$ with dissociation constant K_D, assuming no ligand depletion. There is additional energy coming from nearest-neighbors (Ising) cooperativity C_L. To enumerate the sites on the fibril, and therefore all the configurations, label the sites in the linear array from 1 to N. Introduce two empty sites on either end of the array labeled 0 and $N+1$; this will make the enumeration easier without affecting the total number or energy of each configuration. Then with indicator function s_i taking value 1 if site i is occupied and value 0 if site i is empty, the energy of a configuration is

$$\mathcal{H} = \sum_{i=1}^{N} (\Delta G_L s_i + C_L s_i s_{i+1}). \#(S3)$$

The partition function is the constant of proportionality for the probabilities

$$Z = \sum_{config} \exp\frac{-\mathcal{H}}{k_B T}. \#(S4)$$

Let n_{config} be the number of sites bound for a given configuration. Then the thermodynamic average of the number of sites bound $\langle n \rangle$ can be written as

bioRxiv preprint doi: https://doi.org/10.1101/2025.01.29.635590; this version posted January 30, 2025. The copyright holder for this preprint (which was not certified by peer review) is the author/funder, who has granted bioRxiv a license to display the preprint in perpetuity. It is made available under aCC-BY 4.0 International license.

$$\langle n \rangle = \sum_{config} n_{config} \mathbb{P}(config) = \frac{1}{Z} \sum_{config} n_{config} \exp \frac{-\mathcal{H}}{k_B T} \,.\#(S5)$$

Notice that the Hamiltonian is only a function of the number of sites bound n_{config} and the number of occupied neighboring pairs of sites p_{config}:

$$\mathcal{H} = \Delta G_L n_{config} + C_L p_{config}.\#(S6)$$

So using $h = -\frac{\Delta G_L}{k_B T}$ and $K = -\frac{C_L}{k_B T}$ allows us to write the Boltzmann weight as $\exp\left(h n_{config} + K p_{config}\right)$, the partition function as

$$Z = \sum_{config} \exp\left(h n_{config} + K p_{config}\right) ,\#(S7)$$

and the average number of sites bound as

$$\langle n \rangle = \frac{1}{Z} \sum_{config} n_{config} \exp\left(h n_{config} + K p_{config}\right) .\#(S8)$$

In the same way one can get the average energy by taking the appropriate partial derivative of $\ln Z$, notice that

$$\frac{\partial \ln Z}{\partial h} = \frac{1}{Z} \frac{\partial Z}{\partial h} = \frac{1}{Z} \sum_{config} n_{config} \exp\left(h n_{config} + K p_{config}\right) = \langle n \rangle.\#(S9)$$

We can further simplify by using $L = \frac{[L]}{K_D} = \exp \frac{-\Delta G_L}{k_B T} = \exp h$ (no brackets on the L to indicate non-dimensionalized). Using the Chain Rule from calculus, we finally arrive at

$$\langle n \rangle = \frac{\partial \ln Z}{\partial h} = \frac{\partial L}{\partial h} \frac{\partial \ln Z}{\partial L} = L \frac{\partial \ln Z}{\partial L} \,.\#(S10)$$

So, like much of statistical mechanics, this problem reduces to finding the partition function.

There is a technique from condensed matter physics for finding the partition function of an infinite linear array of interacting particles: the transfer matrix. In our case,

bioRxiv preprint doi: https://doi.org/10.1101/2025.01.29.635590; this version posted January 30, 2025. The copyright holder for this preprint (which was not certified by peer review) is the author/funder, who has granted bioRxiv a license to display the preprint in perpetuity. It is made available under aCC-BY 4.0 International license.

each position on the fibril i can be in one of two states, denoted in Dirac Notation by the kets $|s_i\rangle = \binom{1}{0}$ for a filled site and $|s_i\rangle = \binom{0}{1}$ for an empty site. In this basis, the transfer matrix element $\langle s_{i+1}|T|s_i\rangle$ gives the extra Boltzmann weight in going down the fibril from site s_i to site s_{i+1}. That is,

$$T = \begin{pmatrix} \exp\frac{-(\Delta G_L + C_L)}{k_B T} & \exp\frac{-\Delta G_L}{k_B T} \\ 1 & 1 \end{pmatrix}. \#(S11)$$

The calculations will be easier if we split the Boltzmann weight in going from an empty site to a filled site (or vice-versa) into two equal parts, making the matrix symmetric:

$$T = \begin{pmatrix} \exp\frac{-(\Delta G_L + C_L)}{k_B T} & \exp\frac{-\Delta G_L}{2k_B T} \\ \exp\frac{-\Delta G_L}{2k_B T} & 1 \end{pmatrix}. \#(S12)$$

In terms of L and α_L, the transfer matrix is

$$T = \begin{pmatrix} \alpha_L L & \sqrt{L} \\ \sqrt{L} & 1 \end{pmatrix}. \#(S13)$$

Using the fact that the extra sites s_0 and s_{N+1} appended to each end are empty, several resolutions of the identity, and a proof by induction, we can write the partition function as

$$Z = \langle s_{N+1}|T^{N+1}|s_0\rangle. \#(S14)$$

Since T is Hermitian by virtue of being real and symmetric, the Spectral Theorem of linear algebra guarantees that it has an orthonormal basis of eigenvectors and real eigenvalues:

$$T = \sum_{i=+,-} \lambda_i |i\rangle\langle i|, \#(S15)$$

bioRxiv preprint doi: https://doi.org/10.1101/2025.01.29.635590; this version posted January 30, 2025. The copyright holder for this preprint (which was not certified by peer review) is the author/funder, who has granted bioRxiv a license to display the preprint in perpetuity. It is made available under aCC-BY 4.0 International license.

where λ_+ is the larger eigenvalue with normalized eigenvector $|+\rangle$ and λ_- is the larger eigenvalue with normalized eigenvector $|-\rangle$. Using the orthonormality of this basis, we can write

$$T^{N+1} = \sum_{i=+,-} \lambda_i^{N+1} |i\rangle\langle i| \, , \#(S16)$$

Since $\frac{\lambda_-}{\lambda_+} < 1$, in the limit of large N, we have

$$Z \approx \lambda_+^{N+1} \langle s_{N+1}|+\rangle\langle +|s_0\rangle = \lambda_+^{N+1} v_{+,2}^2 \, , \#(S17)$$

where $v_{+,2} = \langle s_{N+1}|+\rangle = \langle (0 \quad 1)|+\rangle = \langle +|s_0\rangle = \left\langle + \middle| \begin{pmatrix} 0 \\ 1 \end{pmatrix} \right\rangle$ is the second component of the normalized eigenvector with eigenvalue λ_+. $v_{+,2}^2$is of order $\frac{1}{\lambda_+}$, so we get the final simplifications

$$Z \approx \lambda_+^N \#(S18)$$

and

$$\langle n \rangle = L \frac{\partial \ln Z}{\partial L} \approx L \frac{\partial \ln \lambda_+^N}{\partial L} = NL \frac{\partial \ln \lambda_+}{\partial L} = \frac{NL}{\lambda_+} \frac{\partial \lambda_+}{\partial L} \, . \#(S19)$$

The eigenvectors of the matrix T are

$$\lambda_\pm = \frac{\alpha_L L + 1 \pm \sqrt{(\alpha_L L + 1)^2 + 4L}}{2} \, . \#(S20)$$

Plugging the expression for λ_+ into EQ S19 and simplifying, we get

$$\langle n \rangle = N \left(\frac{\alpha_L L - 1 + \sqrt{(\alpha_L L - 1)^2 + 4L}}{2\sqrt{(\alpha_L L - 1)^2 + 4L}} \right) . \#(S21)$$

Assuming that the fibril is long enough that there are no significant edge effects on the many sites in the middle, we have $\langle n \rangle = \langle Nf \rangle = N\langle f_L \rangle$ for the average of the fraction of sites bound with radioligand f_L. Then

bioRxiv preprint doi: https://doi.org/10.1101/2025.01.29.635590; this version posted January 30, 2025. The copyright holder for this preprint (which was not certified by peer review) is the author/funder, who has granted bioRxiv a license to display the preprint in perpetuity. It is made available under aCC-BY 4.0 International license.

$$\langle f_L \rangle = \frac{\alpha_L L - 1 + \sqrt{(\alpha_L L - 1)^2 + 4L}}{2\sqrt{(\alpha_L L - 1)^2 + 4L}} \, , \#(S22)$$

which is the binding isotherm for a saturation assay assuming no ligand depletion.

There is another way to calculate this isotherm from the partition function that will be useful for the case of competition binding assays. For an arbitrary site i, its fraction bound (thermodynamic average over 0 and 1 over all configurations) is

$$\langle s_i \rangle = \frac{1}{Z} \sum_{\substack{s_j \in \{0,1\} \\ j \in \{1,\ldots,N\}}} s_i \exp \frac{-\mathcal{H}}{k_B T} \, , \#(S23)$$

meaning that we evaluate the occupancy of site i (0 for empty and 1 for filled) for each configuration, weight the value by the Boltzmann weight of the configuration, sum, and normalize by the partition function.

Introduce the “pseudo-Pauli matrix”

$$\tilde{\sigma} = \begin{pmatrix} 1 & 0 \\ 0 & 0 \end{pmatrix} , \#(S24)$$

named so because it serves the same purpose as the Pauli matrix $\sigma_z = \begin{pmatrix} 1 & 0 \\ 0 & -1 \end{pmatrix}$ in the theory of the Ising magnet but does not have zero trace. As with σ_z in the Ising magnet, the action of $\tilde{\sigma}$ on the state ket of site i serves to multiply by the state occupancy:

$$\tilde{\sigma}|s_i\rangle = \begin{cases} \begin{pmatrix} 1 & 0 \\ 0 & 0 \end{pmatrix} \begin{pmatrix} 1 \\ 0 \end{pmatrix} = \begin{pmatrix} 1 \\ 0 \end{pmatrix} = 1 * |s_i\rangle \; if \; filled \\ \begin{pmatrix} 1 & 0 \\ 0 & 0 \end{pmatrix} \begin{pmatrix} 0 \\ 1 \end{pmatrix} = \begin{pmatrix} 0 \\ 0 \end{pmatrix} = 0 * |s_i\rangle \; if \; empty. \end{cases} \#(S25)$$

By an argument like the one leading to the expression for the partition function in EQ S14, we can write EQ S23 for the average $\langle s_i \rangle$ as

$$\langle s_i \rangle = \frac{\langle s_{N+1} | T^{N+1-i} \, \tilde{\sigma} \, T^i | s_0 \rangle}{Z} \, . \#(S26)$$

bioRxiv preprint doi: https://doi.org/10.1101/2025.01.29.635590; this version posted January 30, 2025. The copyright holder for this preprint (which was not certified by peer review) is the author/funder, who has granted bioRxiv a license to display the preprint in perpetuity. It is made available under aCC-BY 4.0 International license.

If the site of interest is in the middle of a sufficiently long fibril, then both i and $N+1-i$ will be large, so $T^i \approx \lambda_+^i|+\rangle\langle+|$ and $T^{N+1-i} \approx \lambda_+^{N+1-i}|+\rangle\langle+|$. Then

$$\langle s_i\rangle \approx \frac{\langle s_{N+1}|\lambda_+^{N+1-i}|+\rangle\langle+|\tilde{\sigma}\lambda_+^i|+\rangle\langle+|s_0\rangle}{Z} = \frac{\lambda_+^{N+1}\langle s_{N+1}|+\rangle\langle+|\tilde{\sigma}|+\rangle\langle+|s_0\rangle}{Z} \approx \langle+|\tilde{\sigma}|+\rangle, \#(S27)$$

since $Z \approx \lambda_+^{N+1}\langle s_{N+1}|+\rangle\langle+|s_0\rangle$ by EQ S16. Evaluating this matrix element is equivalent to squaring the first component of the normalized eigenvector of the larger eigenvalue and gives the same expression as EQ S22.

We can use the same approach to derive the equilibrium isotherm for a competition experiment. In this case, each site can have a radioligand bound, have an inhibitor (test ligand) bound, or be empty. This situation describes a 3-state system: in Dirac notation, the kets become $|s_i\rangle = \begin{pmatrix}1\\0\\0\end{pmatrix}$ for radioligand binding, $|s_i\rangle = \begin{pmatrix}0\\1\\0\end{pmatrix}$ for inhibitor binding, and $|s_i\rangle = \begin{pmatrix}0\\0\\1\end{pmatrix}$ for empty. There are similar parameters involving the inhibitor as with the radioligand, as described in the main text: $\Delta G_I = -k_BT\ln\frac{[I]}{K_I}$ added for each site bound with inhibitor at free concentration $[I]$, $C_I = -k_BT\ln\alpha_I$ added for every pair of neighboring sites both bound with inhibitor, and $C_{L-I} = -k_BT\ln\chi$ for each pair of neighboring sites bound with different species. The transfer matrix is then

bioRxiv preprint doi: https://doi.org/10.1101/2025.01.29.635590; this version posted January 30, 2025. The copyright holder for this preprint (which was not certified by peer review) is the author/funder, who has granted bioRxiv a license to display the preprint in perpetuity. It is made available under aCC-BY 4.0 International license.

$$T = \begin{pmatrix} \exp\frac{-(\Delta G_L + C_L)}{k_B T} & \exp\frac{-\left(\frac{1}{2}\Delta G_L + \frac{1}{2}\Delta G_I + C_{L-I}\right)}{k_B T} & \exp\frac{-\Delta G_L}{2k_B T} \\ \exp\frac{-\left(\frac{1}{2}\Delta G_L + \frac{1}{2}\Delta G_I + C_{L-I}\right)}{k_B T} & \exp\frac{-(\Delta G_I + C_I)}{k_B T} & \exp\frac{-\Delta G_I}{2k_B T} \\ \exp\frac{-\Delta G_L}{2k_B T} & \exp\frac{-\Delta G_I}{2k_B T} & 1 \end{pmatrix}$$

$$= \begin{pmatrix} \alpha_L L & \chi\sqrt{LI} & \sqrt{L} \\ \chi\sqrt{LI} & \alpha_I I & \sqrt{I} \\ \sqrt{L} & \sqrt{I} & 1 \end{pmatrix}, \#(S28)$$

where $I = \frac{[I]}{K_I}$. We have again broken the Boltzmann weight contribution for going from one site to one with a different occupancy (off-diagonals) in two so that the matrix will be symmetric (and therefore be diagonalizable with real eigenvalues). There will again be two appended empty sites: $|s_0\rangle = |s_{N+1}\rangle = \begin{pmatrix} 0 \\ 0 \\ 1 \end{pmatrix}$ The partition function is still $Z = \langle s_{N+1}|T^{N+1}|s_0\rangle$. Assuming T is non-degenerate, call the largest of its 3 eigenvalues λ_1, with normalized eigenvector $|1\rangle$. We can similarly find the average fraction of sites bound with radioligand as $\frac{L}{\lambda_1}\frac{\partial \lambda_1}{\partial L}$ (EQ S19) or $\langle 1|\tilde{\sigma}|1\rangle$ (EQ S27), using a new 3×3 matrix $\tilde{\sigma} = \begin{pmatrix} 1 & 0 & 0 \\ 0 & 0 & 0 \\ 0 & 0 & 0 \end{pmatrix}$.

While the saturation assay gave a 2-state system, a 2×2 transfer matrix, and 2 eigenvalues easily found with the quadratic formula, this setup of the competition assay requires finding the largest of 3 eigenvalues of an arbitrary real symmetric matrix. Assuming every site is bound is one way to reduce this problem to the tractable 2-state system. The transfer matrix looks like

bioRxiv preprint doi: https://doi.org/10.1101/2025.01.29.635590; this version posted January 30, 2025. The copyright holder for this preprint (which was not certified by peer review) is the author/funder, who has granted bioRxiv a license to display the preprint in perpetuity. It is made available under aCC-BY 4.0 International license.

$$T \approx \begin{pmatrix} \alpha_L L & \chi\sqrt{LI} & 0 \\ \chi\sqrt{LI} & \alpha_I I & 0 \\ 0 & 0 & 0 \end{pmatrix}. \#(S29)$$

We can see that this approximation is valid at concentrations of radioligand and inhibitor high enough, given their molecular parameters, so that $\alpha_L L$, $\alpha_I I$, and $\chi\sqrt{LI}$ are each much larger than $\sqrt{L}$, $\sqrt{I}$, and 1. Even if we do not know the molecular parameters K_D, K_I, α_L, α_I, and χ *a priori* (hence the experiment), we can always increase the free ligand concentrations (up to solubility limits) so that this approximation works. In this limit, 0 is obviously an eigenvalue, and the other two are $\frac{\alpha_L L + \alpha_I I \pm \sqrt{(\alpha_L L - \alpha_I I)^2 + 4\chi^2 LI}}{2}$, so the largest is the non-zero one with the plus sign. Then by either method, we can get the expression for $\langle f_L \rangle$ given by EQ 6 in the main text.

Another way to simplify the transfer matrix ins EQ S28 is to assume that there is one common cooperativity between all pairs of molecular species: $\alpha_L = \alpha_I = \chi = \alpha$ means

$$T = \begin{pmatrix} \alpha L & \alpha\sqrt{LI} & \sqrt{L} \\ \alpha\sqrt{LI} & \alpha I & \sqrt{I} \\ \sqrt{L} & \sqrt{I} & 1 \end{pmatrix}. \#(S30)$$

While the experimenter does not have control over the system to ensure this condition is always met, an approximation along the lines of $\alpha_L \approx \alpha_I \approx \chi$ is reasonable if both species are very similar. The approximation is always exact if the two species are "hot" (radiolabeled) and "cold" versions of the same molecule, which the experimenter can control. Unlike in the condition before, we do not need to have every site be filled, meaning we can run the experiment with arbitrary radioligand and inhibitor concentrations. The transfer matrix in EQ S30 is just simple enough that we can find its

bioRxiv preprint doi: https://doi.org/10.1101/2025.01.29.635590; this version posted January 30, 2025. The copyright holder for this preprint (which was not certified by peer review) is the author/funder, who has granted bioRxiv a license to display the preprint in perpetuity. It is made available under aCC-BY 4.0 International license.

largest eigenvalue: $\lambda_1 = \frac{1+\alpha(L+I)+\sqrt{1+2(2-\alpha)(L+I)+\alpha^2(L+I)^2}}{2}$. Using the approach of $\frac{L}{\lambda_1}\frac{\partial\lambda_1}{\partial L}$ (EQ S19) or $\langle 1|\tilde{\sigma}|1\rangle$ (EQ S27), we then have

$$\langle f_L\rangle = \frac{L}{L+I}\frac{\alpha(L+I)-1+\sqrt{\alpha^2I^2+2I(\alpha^2L-\alpha+2)+(\alpha L-1)^2+4L}}{2\sqrt{\alpha^2I^2+2I(\alpha^2L-\alpha+2)+(\alpha L-1)^2+4L}}\,.\#(S31)$$

This function has all the expected limits. First, filling all the sites by making $\alpha \gg 1$ (equivalently, making the transfer matrix in the derivation into $T = \begin{pmatrix} \alpha L & \alpha\sqrt{LI} & 0 \\ \alpha\sqrt{LI} & \alpha I & 0 \\ 0 & 0 & 0 \end{pmatrix}$) leads to

$$\langle f_L\rangle = \frac{L}{L+I}\,.\#(S32)$$

We also get this limit by first imposing the requirement that every site is filled, then deriving EQ 9 in the main text, and then imposing $\alpha_L = \alpha_I = \chi = \alpha$. The meaning of this limit is that each site can be bound with radioligand (Boltzmann weight $\alpha^2 L$) or inhibitor ($\alpha^2 I$), and the constant cooperativity makes the binding across sites independent, giving $\langle f_L\rangle = \frac{\alpha^2 L}{\alpha^2 L+\alpha^2 I} = \frac{L}{L+I}$. We also get the halfway point when $I = L$, or

$$IC_{50} = K_I\frac{[L]}{K_D}\,,\#(S33)$$

which agrees with the main text's EQ 7 when $\alpha_L = \alpha_I$.

The next limit to check is $\alpha = 1$, meaning there is no cooperativity between any pair of molecular species. Then

$$\langle f_L\rangle = \frac{L}{L+I+1}\,,\#(S34)$$

bioRxiv preprint doi: https://doi.org/10.1101/2025.01.29.635590; this version posted January 30, 2025. The copyright holder for this preprint (which was not certified by peer review) is the author/funder, who has granted bioRxiv a license to display the preprint in perpetuity. It is made available under aCC-BY 4.0 International license.

which is what we would expect for competitive inhibition of independent receptors or enzymes operating with Michaelis-Menten kinetics. The halfway point is exactly the Cheng-Prusoff Equation

$$IC_{50} = K_I\left(1 + \frac{[L]}{K_D}\right). \#(S35)$$

Finally, adding no inhibitor to the system means setting $I = 0$, leading to the saturation assay binding isotherm given by EQ 5.

For the case of every site being bound, we can derive the biphasic behavior for $\beta > 3$. We define a biphasic isotherm as one with 3 inflection points on the logarithmic axis. With $I = \frac{[I]}{IC_{50}}$ and $y = \log_{10} I$, an inflection point occurs whenever $\frac{d^2\langle f_L\rangle}{dy^2} = 0$ for a given β value. Using the expression for $\langle f_L\rangle$ in EQ 9 in the main text,

$$\frac{d^2\langle f_L\rangle}{dy^2} = \frac{\beta(\ln 10)^2 I(I-1)(I^2 + 2(2-\beta)I + 1)}{((I-1)^2 + 4\beta I)^{5/2}}. \#(S36)$$

Since $I > 0$ the denominator is always positive, and the inflection points are given by the roots of the numerator. We never get the root at $I = 0$ and always get the root at $I = 1$ (the IC_{50}). The quadratic in the numerator has roots at $\beta - 2 \pm \sqrt{(\beta-2)^2 - 1}$. These roots are real and positive only for $\beta > 3$. The case of $\beta = 3$ just increases the multiplicity of the root at 1. If the cooperativity energies C_L, C_I, and C_{L-I} do not depend on temperature, then the phase transition at $\beta = 3$ happens at temperature

$$T = \frac{-(2C_{L-I} - C_L - C_I)}{k_B \ln 3}. \#(S37)$$

Lastly, we derive the average mixing for the competition assay as follows. First, allow for a site to have either molecular species or nothing bound. Use the indicator function s_i for whether a site has radioligand bound and the indicator function t_i for

bioRxiv preprint doi: https://doi.org/10.1101/2025.01.29.635590; this version posted January 30, 2025. The copyright holder for this preprint (which was not certified by peer review) is the author/funder, who has granted bioRxiv a license to display the preprint in perpetuity. It is made available under aCC-BY 4.0 International license.

whether a site has inhibitor bound. Since a site cannot have both species bound at the same time, the possible states for the system described by $\binom{s_i}{t_i}$ are $\binom{0}{0}$ for empty, $\binom{1}{0}$ for radioligand bound, and $\binom{0}{1}$ for inhibitor bound, with $\binom{1}{1}$ not allowed. This scheme describes the same 3-state system as before, and the extra empty sites appended to either end of the fibril means $s_0 = s_{N+1} = t_0 = t_{N+1} = 0$. The mixing across all sites of a given configuration is the number of radioligand sites followed by inhibitor sites plus the number of inhibitor sites followed by radioligand sites: $\frac{1}{N}\sum_{i=1}^{N}(s_i t_{i+1} + t_i s_{i+1})$. The energy (Hamiltonian) for a given configuration is

$$\mathcal{H} = \sum_{i=1}^{N}\left(\Delta G_L s_i + \Delta G_I t_i + C_L s_i s_{i+1} + C_I t_i t_{i+1} + C_{L-I}(s_i t_{i+1} + t_i s_{i+1})\right), \#(S38)$$

the partition function is

$$Z = \sum_{\substack{\binom{s_i}{t_i}\in\left\{\binom{0}{0},\binom{1}{0},\binom{0}{1}\right\} \\ i\in\{1,\dots,N\}}} \exp\frac{-\mathcal{H}}{k_B T}, \#(S39)$$

and the (thermodynamic) average mixing is

$$\langle Mixing\rangle = \frac{1}{Z}\sum_{\substack{\binom{s_i}{t_i}\in\left\{\binom{0}{0},\binom{1}{0},\binom{0}{1}\right\} \\ i\in\{1,\dots,N\}}} \frac{1}{N}\left(\sum_{i=1}^{N}(s_i t_{i+1} + t_i s_{i+1})\right)\exp\frac{-\mathcal{H}}{k_B T}. \#(S40)$$

If we define $D = -\frac{C_{L-I}}{k_B T}$, so $\chi = \exp\frac{-C_{L-I}}{k_B T} = e^D$, then we can find the average mixing as

$$\langle Mixing\rangle = \frac{1}{N}\frac{\partial \ln Z}{\partial D} = \frac{1}{N}\frac{\partial \chi}{\partial D}\frac{\partial \ln Z}{\partial \chi} = \frac{\chi}{N}\frac{\partial \ln Z}{\partial \chi}. \#(S41)$$

bioRxiv preprint doi: https://doi.org/10.1101/2025.01.29.635590; this version posted January 30, 2025. The copyright holder for this preprint (which was not certified by peer review) is the author/funder, who has granted bioRxiv a license to display the preprint in perpetuity. It is made available under aCC-BY 4.0 International license.

If we use the assumption that all the sites are bound with something (radioligand or inhibitor), then we get the simplification of $Z \approx \lambda_+^N$, where $\lambda_+ = \frac{\alpha_L L + \alpha_I I + \sqrt{(\alpha_L L - \alpha_I I)^2 + 4\chi^2 LI}}{2}$.

Then

$$\langle Mixing \rangle \approx \frac{\chi}{N}\frac{\partial \ln \lambda_+^N}{\partial \chi} = \frac{\chi}{\lambda_+}\frac{\partial \lambda_+}{\partial \chi} \,. \#(S42)$$

After simplifying with $IC_{50} = EC_{50,I}\frac{[L]}{EC_{50,L}} = \frac{K_I}{\alpha_I}\frac{\alpha_L}{K_D}[L]$ (from EQ 5 and EQ 7 in the main text) and $\beta = \frac{\chi^2}{\alpha_L \alpha_I}$, we get

$$\langle Mixing \rangle = \frac{4\beta \frac{[I]}{IC_{50}}}{\sqrt{\left(1 - \frac{[I]}{IC_{50}}\right)^2 + 4\beta \frac{[I]}{IC_{50}}}\left(\sqrt{\left(1 - \frac{[I]}{IC_{50}}\right)^2 + 4\beta \frac{[I]}{IC_{50}}} + 1 + \frac{[I]}{IC_{50}}\right)} \,, \#(S43)$$

which we plot above as a function of $x = \log_{10}\frac{[I]}{IC_{50}}$ for different values of β. The mixing is always the best at $[I] = IC_{50}$, and as β increases, the range of inhibitor concentrations for good mixing increases. The mixing will only be perfect (equal to 1) in the limit of infinite β, which can happen at zero temperature, at which case the entropy of mixing does not contribute to the free energy.
